# Retraction Note: Histone acetyltransferase PCAF accelerates apoptosis by repressing a GLI1/BCL2/BAX axis in hepatocellular carcinoma

**DOI:** 10.1038/s41419-026-09338-0

**Published:** 2026-10-08

**Authors:** X. Gai, K. Tu, C. Li, Z. Lu, L. R. Roberts, X. Zheng

**Affiliations:** 1 Department of Hepatobiliary Surgery, the First Affiliated Hospital of Xi’an Jiaotong University, Xi’an, Shaanxi 710061 China; 2 Division of Gastroenterology and Hepatology, Mayo Clinic College of Medicine, Rochester, MN 55905 USA

Retraction Note to: *Cell Death & Disease* https://doi.org/10.1038/cddis.2015.76, published online 09 April 2015

The Editor-in-chief has retracted this article. After publication, concerns were raised regarding highly image similarities within the article and with other publications from the same authors. Specifically:

- Fig.1A, β-actin appears to be highly similar to Fig.1C, β-actin and Fig.4C, β-actin in this article and to Fig.5B, GAPDH in [1, 2]
- Fig.1C, GLI1 and Fig.6B, Fibronectin (Scr siRNAs) [3] appear to be highly similar
- Fig.1C, PTCH1 and Fig.6B, Fibronectin (SNAI1 siRNAs) [3] (180-degree flipped) appear to be highly similar
- Fig.1D, GLI1 appears to be highly similar to Fig.2N, Acetylated GlI1 in this article and to Fig.5B, FBXW7 in [1]
- Fig.3B, Bcl-2 appears to be highly similar to Fig.3D, N-cadherin and Fig.3D, Fibronectin in [3]
- Fig.4A, PLC/PRF/5 Vector appears to be highly similar to Fig.5B, PLC/PRF/5 Vector (5-FU: 0 μM) in this article and to Fig.3C, miR-control in [4] and Fig.4C (left panel) in [5]
- Fig.4A, PLC/PRF/5 PCAF appears to be highly similar to Fig.5B, PLC/PRF/5 PCAF (5-FU: 0 μM) in this article and to Fig.3C, Huh7 Vector in [5]
- Fig.4C, B-actin and Fig.5B, GAPDH [1] appear to be highly similar
- Fig.5B, PLC/PRF/5 Vector (5-FU: 0 μM) appears to be highly similar to Fig.3C, miR-control in [4], and to Fig.3C, Huh7 Vector, Fig.4C (left panel) and Fig.3C, Huh7 Vector in [5]
- Fig.5B, PLC/PRF/5 Vector (5-FU: 0 μM) appears to be highly similar to Fig.3C, miR-control in [4] and Fig.4C (left panel) in [5]
- Fig.5B, PLC/PRF/5 PCAF (5-FU : 0 μM) and Fig.3C, Huh7 Vector [5] appear to be highly similar
- Fig.7C, BAX (PLC/PRF/5 Vector) and Fig.8, N-cadherin (Huh7 Vector) [3] appear to be highly similar
- Fig.7C, BAX (PLC/PRF/5 PCAF) and Fig.8, CtBP2 (Huh7 CtBP2) [3] appear to be highly similar

The authors were given the opportunity to address the concerns raised; however, they did not respond to the Publisher’s correspondence. Due to the number of concerns, the Editor-in-chief no longer has confidence in the results and conclusions of this article.

The authors did not respond to correspondence from the Publisher about this retraction.
